# Genome-Wide Screening of Transposable Elements in the Whitefly, *Bemisia tabaci* (Hemiptera: Aleyrodidae), Revealed Insertions with Potential Insecticide Resistance Implications

**DOI:** 10.3390/insects13050396

**Published:** 2022-04-19

**Authors:** Marwa Zidi, Khouloud Klai, Johann Confais, Benoît Chénais, Aurore Caruso, Françoise Denis, Maha Mezghani Khemakhem, Nathalie Casse

**Affiliations:** 1 Laboratory of Biochemistry and Biotechnology (LR01ES05), Faculty of Sciences of Tunis, University of Tunis El Manar, Tunis 1068, Tunisia; marwa.zidi@fst.utm.tn (M.Z.); khouloud.klai@fst.utm.tn (K.K.); 2 Laboratory of Biology of Organisms, Stress, Health, Environment (BiOSSE), Le Mans University, 72085 Le Mans, France; bchenais@univ-lemans.fr (B.C.); aurore.caruso@univ-lemans.fr (A.C.); fdenis@univ-lemans.fr (F.D.); 3 Université Paris-Saclay, INRAE, URGI, 78026 Versailles, France; johann.confais@inrae.fr; 4 Université Paris-Saclay, INRAE, BioinfOmics, Plant bioinformatics facility, 78026 Versailles, France

**Keywords:** transposable elements, TE age, *Bemisia tabaci*, insecticide resistance

## Abstract

**Simple Summary:**

Transposable elements (TEs) are mobile DNA sequences hosted in the genomes of various organisms. These elements have the ability to mediate regulatory changes, which can result in changes in gene expression. *Bemisia tabaci* is an important agricultural pest that has been linked to several cases of insecticide resistance. In this study, we conducted a genome-wide screening of TEs in the *B. tabaci* genome using bioinformatics tools. Results revealed a total of 1,292,393 TE copies clustered into 4872 lineages. The TE insertion site analysis revealed 94 insertions within or near defensome genes.

**Abstract:**

Transposable elements (TEs) are genetically mobile units that move from one site to another within a genome. These units can mediate regulatory changes that can result in massive changes in genes expression. In fact, a precise identification of TEs can allow the detection of the mechanisms involving these elements in gene regulation and genome evolution. In the present study, a genome-wide analysis of the Hemipteran pest *Bemisia tabaci* was conducted using bioinformatics tools to identify, annotate and estimate the age of TEs, in addition to their insertion sites, within or near of the defensome genes involved in insecticide resistance. Overall, 1,292,393 TE copies were identified in the *B. tabaci* genome grouped into 4872 lineages. A total of 699 lineages were found to belong to Class I of TEs, 1348 belong to Class II, and 2825 were uncategorized and form the largest part of TEs (28.81%). The TE age estimation revealed that the oldest TEs invasion happened 14 million years ago (MYA) and the most recent occurred 0.2 MYA with the insertion of Class II TE elements. The analysis of TE insertion sites in defensome genes revealed 94 insertions. Six of these TE insertions were found within or near previously identified differentially expressed insecticide resistance genes. These insertions may have a potential role in the observed insecticide resistance in these pests.

## 1. Introduction

The whitefly *Bemisia tabaci* (Hemiptera: Aleyrodidae) is a serious global pest of vegetable and ornamental crops [1,2]. It feeds on over 1000 ornamental and vegetable plant species from 74 different families including the Asteraceae (sunflower, aster flowers), Fabaceae (acacia., lotus), and Solanaceae (pepper, tomato) that are among the most infested plants [3]. Moreover, the whitefly is a well-known supervector of over 300 plant viruses. namely the Tomato chlorosis virus, the Cucumber vein yellowing virus, and the Tomato yellow leaf curl virus, which is one of the most damaging viruses that infects tomato crops around the world [4].

Furthermore. *B. tabaci* has developed resistance to nearly all insecticides used for its control, including organophosphates, carbamates, pyrethroids, neonicotinoids, and thiadiazine [5]. Recently, gene annotation of the whitefly genome allowed the identification of 647 defensome genes involved in insecticide resistance, including 130 cytochrome P450 (CYPs), 81 UDP-glucuronosyltransferases (UGTs), 22 glutathione S-transferases (GSTs), 50 ATP-Binding Cassette (ABC) transporters, 51 carboxylesterases (CCEs), 202 phosphatidylethanolamine binding proteins (PEBP), and 111 cathepsins [6]. The annotation of transposable elements (TEs) may be useful to derive a better understanding of the origins of insecticide resistance. In fact, TEs are considered as powerful evolutionary drivers in all living organisms since they play an important role in genomic evolution and environmental adaptation of species [7]. 

TEs are known as “jumping” DNA sequences allowing several insertions into host genomes and generating multiple sites for chromosomal rearrangements, which may have either deleterious or beneficial consequences [8,9,10]. Furthermore, TEs can confer selective advantages through their insertion sites by either enhancing or suppressing gene expression or being domesticated as a new host gene [11,12,13,14]. Several studies showed that TEs insertions into specific genes were linked to insecticide resistance. For example, in the *Drosophila melanogaster*, it was demonstrated that the insertion of an *Accord* transposable element into the 5′ end of the Cyp6g1 gene leads to its overexpression and explains the conferred resistance to Dichloro-diphenyl-trichloroethane [15,16]. Moreover, the insertion of a *Doc* element into the second exon of the CG10618 gene called *CHKov1* is responsible for organophosphate resistance [17]. More recently, several TE insertions near genes were linked to xenobiotics and oxidative stress resistance in *D. melanogaster* [18]. In the tobacco budworm, *Heliothis* *virescens*, resistance to the *Bacillus thuringiensis* (Bt) toxin Cry1Ac was linked to the insertion of TEs into a cadherin-superfamily gene [19]. Klai et al. (2020) described nine TE insertions in exons and introns of CyP450, GST, and ABC genes in the genome of the cotton bollworm *Helicoverpa armigera,* but the implications of these insertions in insecticide resistance need to be further investigated [20].

TEs are classified into two main classes based on structural and biological features, according to the most improved classification systems [21,22]. The Class I TEs use the replicative (copy and paste) mechanism, whereas the Class II TEs use the conservative (cut and paste) mechanism to move from a position to another in a host genome [23]. The Class I TEs, also known as retrotransposons, replicate via an RNA intermediate that is retrotranscribed to cDNA and integrated in a new genomic site using the Reverse Transcriptase (RT), RNAseH, and Integrase. According to their structure, this class of elements is divided into seven orders, namely Long Terminal Repeat (LTR), *Dictyostelium* Intermediate Repeat Sequence (DIRS), Penelope (PLE), Long INterspersed Element (LINE), Short INterspersed Element (SINE), Terminal Repeat retrotransposons In Miniature (TRIM), and Large Retrotransposon Derivative (LARD). With few exceptions, Class II TEs, or DNA transposons, transpose by excision and integration of the element in a new genomic position via the enzymatic activities of their transposase. This class is subdivided into five orders, namely Terminal Inverted Repeats (TIR), Miniature Inverted-repeat Transposable Elements (MITE), Crypton, Helitron, and Maverick. Each order is further subdivided into superfamilies and families depending on their sequence similarities [21,24]. Currently, the genomic survey of TEs has become easier due to the availability of a wide variety of bioinformatics tools developed for TE annotation [25,26].

In this work, a genome-wide analysis of the Hemipteran pest *B. tabaci* was conducted using bioinformatics tools to identify and annotate TEs, in addition to their insertion sites, within or in the vicinity of the defensome genes involved in insecticide resistance.

## 2. Materials and Methods

### 2.1. Supporting Data

The MEAM1/B *B. tabaci* genome of 615 Mb available in the NCBI database (assembly ASM185493v1) was used to identify the TE content of this insect genome and TE insertions in connection with the defensome genes involved in insecticide resistance. This genome was sequenced by Chen et al. (2016) using both Illumina short reads and PacBio long reads approaches, then assembled into 19,751 scaffolds with a 3,232,964 kbp N50 length [6].

### 2.2. TE Identification

The identification of *B. tabaci* TEs was conducted using the REPET v3.0 package with default parameters [27,28], which integrates two main pipelines: TEdenovo for repeats identification and TEannot for their annotation. TEdenovo analysis is based on three main steps. First, the *B. tabaci* genome was cut into batches in order to be compared to itself by the “all against all” method using BLASTER. Second, the detected repetitive High Scoring Pairs were grouped into clusters of repeats using GROUPER and RECON, which are clustering programs specific for interspersed repeats. Then, the results were combined and redundancy was eliminated in order to create a multiple alignment for each cluster from which a consensus sequence was produced. Finally, the consensuses were categorized based on TE characteristics (LTR, TIR, polyA tail, Open Reading Frame, etc.) and their similarities with reference TEs from the Repbase updated database V26.10 [29], Pfam databases [30], and HMM profiles. Finally, a classification step was carried out by PASTEC [31] based on the classification system described by Wicker [21] and a library of non-redundant classified consensus sequences was built.

### 2.3. TE Annotation

TEannot, which includes BLASTER, RepeatMasker, and CENSOR was used to annotate the *B. tabaci* genome with the consensus library generated by TEdenovo. To eliminate false-positive matches and increase the quality of the TE annotation, a double round of TEannot [32] was performed. The first round of TEannot was run to identify the consensuses that annotated at least one Full Length Copy (FLC) (fragmented and unfragmented annotation aligned over more than 95% of the consensus TE sequence). The second was undertaken to increase the TE annotation quality of the sequences by using consensuses that annotated at least one FLC [32]. All of the TE consensus sequences were manually verified to characterize structural features and remove artifactual chimeras and duplications.

### 2.4. Estimation of TEs’ Age Distribution

The insertion and deletion rates of TE families were estimated using the TE package version 0.3-0 implemented in R version 4.0.3 and based on statistical models proposed by Dai et al. (2018) [33]. This consists of an improved estimate of the age distribution that accounts for random mutations, and an adjustment by the deletion rate, to calculate the insertion rate.

### 2.5. TE Insertion Sites

The bedtools-closest tool v2.26.0 [34,35] was used to look for possible TE insertions within the genes (from transcription start site to stop codon) and 2 kb up and downstream [36] by combining TEs’ Generic File Format annotations from TEannot with those of the genes obtained from NCBI database. This program looks for features in the genome that are close to and/or overlap with TE copies. The TE insertion locations in the genes, their orientations, and the distance between genes and TEs were used to classify the insertions. Then, we searched for TE insertions that may be involved in insecticide resistance using Chen et al. 2016’s annotated defensome genes associated with detoxification and insecticide resistance in *B. tabaci* in both susceptible and resistant populations in response to insecticide treatment [6]. These genes were downloaded from the NCBI website (https://www.ncbi.nlm.nih.gov/data-hub/gene/table/taxon/7038/?utm_source=gquery&utm_medium=referral, accessed on 16 March 2021 (File S2).

## 3. Results 

### 3.1. Annotation of TEs in the B. tabaci Genome

The screening of TEs in the genome of *B. tabaci* using the REPET package led to the identification of 1,327,236 putative repeats. After removal of sequences corresponding to satellites and poorly supported sequences without a TE hallmark, 1,292,393 copies were recovered, representing 51% of the whole genome sequence. These elements were clustered into 4872 lineages, of which 699 belong to Class I of TEs, 1348 belong to Class II, and 2825 were uncategorized even though they have TE characteristics (Table 1 and Table S1). The majority of these lineages were represented by at least one FLC, i.e., 27,599 sequences that cover more than 95% of the consensus of each lineage (File S1). The Class I lineages are represented by 73,223 TE copies covering 5.7% of the whole *B. tabaci* genome sequence. The elements of this class were classified into six orders based on their structural characteristics (Table 1).

The LINE order gathers the largest number of the Class I element with 43,262 TE copies and revealed a broad diversity, represented by at least 12 different superfamilies (Table 1). The *RTE* superfamily represents the vast majority of the LINE elements with 14,232 copies (211 FLC) clustered in 34 lineages, followed by the *Jockey* superfamily with 4909 copies (202 FLC) clustered in 87 lineages, indicating high diversity within this superfamily (Table 1).

Regarding the LTR order, the most abundant elements belong to the *Gypsy* and *Copia* superfamilies, which have nearly identical copy numbers of 3797 copies (147 FLC) and 3726 copies (99 FLC), respectively (Table 1). These copies are organized into 64 lineages for *Gypsy* and 56 lineages for *Copia*. The *Gypsy* superfamily showed greater variability among its elements compared to the *Copia* superfamily. The Class II lineages are represented by 213,958 TE copies, which cover 16.55% of the total genome sequence of *B. tabaci* (Table 1). Structural properties of the elements in this class, such as terminal repeats, target site duplication, and characteristics of the protein coding domains, were used to distribute them into five different orders, namely Crypton, Helitron, MITE, Maverick, and TIR (Table 1).

The TIR order includes the largest number of Class II element copies, i.e., 121,068 TE copies, indicating a wide range of variation, with at least 12 different superfamilies (Table 1). The distribution of the elements in this order showed that the *hAT* superfamily has 5797 copies (110 FLC) clustered in 35 lineages, followed by the *Tc1-Mariner* superfamily, which has 5038 copies (407 FLC) clustered in 52 lineages (Table 1). The *Mutator* and *Academ* superfamilies each have a large number of copies with 1640 (124 FLC) and 1097 (42 FLC) copies, respectively (Table 1). These copies are divided into 46 *Mutator* lineages and nine *Academ* lineages. All of these superfamilies show substantial variability among their copies, with the exception of the *Academ* superfamily, which has homogeneous copies.

**Figure 1:**
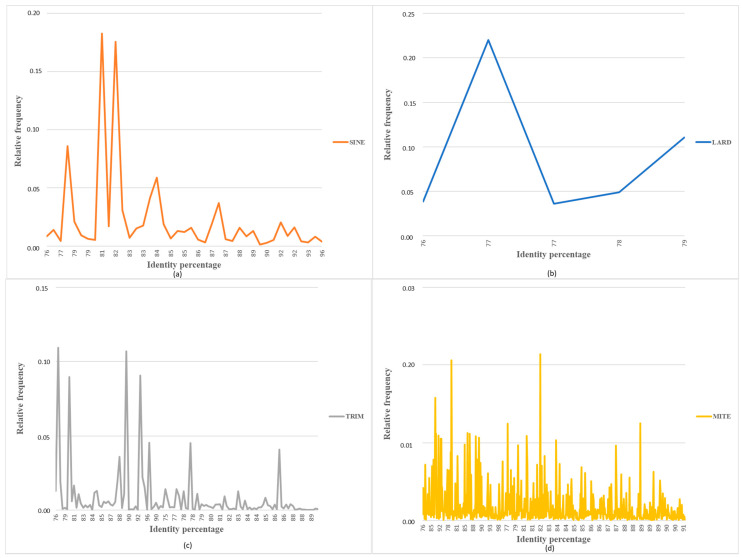
Distribution of sequence identity values between mTE copies and mTE sequences with at least one FLC. The relative frequencies per percentage of identity of (**a**) Short Interspersed Nuclear Elements (SINEs), (**b**) large retrotransposon derivatives, (**c**) terminal repeat retrotransposons in miniature (TRIMs), and (**d**) Miniature Inverted Transposable Element (MITE) orders are represented in different colors.

The miniature Transposable Elements (mTEs), including SINEs and TRIM belonging to Class I, and MITEs from Class II, are present with a high number of copies. The SINE and the TRIM are represented by 3019 copies organized into 42 lineages and 1142 copies divided to 117 lineages, respectively (Table 1). Regarding the LARD elements, they include 859 copies, which belong to six different lineages and the LARDs showed a homogeneous distribution (Figure 1, Table 1). MITEs have the highest copy number. i.e., 84,436 copies, clustered in 564 lineages (Figure 1, Table 1), and the group of MITEs showed the greatest variability between copies among all the mTEs identified.

Finally, the vast majority of the identified TEs could not be classified and showed no obvious similarities with reference TEs from Repbase or Pfam and HMM profiles, so they were labeled as UnCategorized (UnCats). These UnCats were divided into 2825 lineages, which covered approximately 28.81% of the genomic assembly and 77.78% of the covering repeatome. 

### 3.2. Estimation of TEs Age Distribution

**Figure 2:**
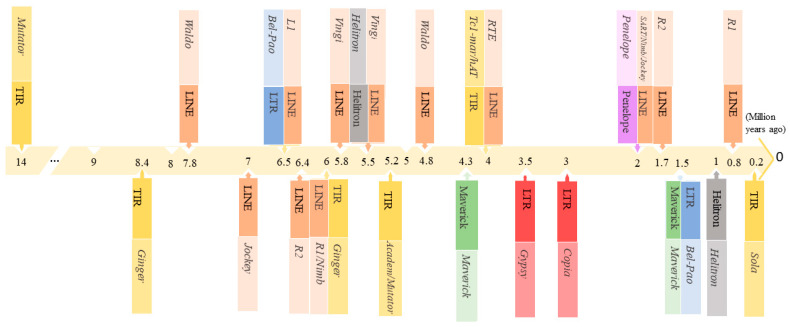
Timeline of the TE age distribution in the *B. tabaci* genome. The TE labels represent the peak of TE activity bursts.

Age estimation of the TEs revealed a varied age distribution, according to the superfamilies and dating back to nearly 14 million years ago (MYA) (Figure 2 and Figure S1). The first invasion of the *B. tabaci* genome began with the insertion of *Mutator* elements belonging to the TIR order, followed by *Ginger* elements around 8.4 MYA. From 5.8 to 7.8 MYA, 12 invasions belonging to multiple families of the LINE order occurred, in addition to a new invasion by elements of the *Ginger* family (TIR order, 6 MYA) and the appearance of *Bel-Pao* elements of the LTR order (6.5 MYA). *Helitron* and the LINE-*Vingi* emerged 5.5 MYA, and the TIRs TE *Academ* and *Mutator* also arose in the same period (5.2 MYA). After the first LINE-*Waldo* wave, which happened 7.8 MYA, a second LINE-*Waldo* wave occurred 4.8 MYA. Then, between 4 and 4.8 MYA, many transposition events of *Tc1-mariner*, *hAT*, *RTE*, and *Maverick* took place. After that, *Copia* and *Gypsy* invasions happened, followed by LINE-*SART*. LINE-*Nimb* and *Jockey* from the LINE order, and Penelope elements, occurred at 2 MYA. Six waves of TEs invasions have been detected since 1.7 MYA, with two belonging to *Maverick* and *Bel-Pao* occurring simultaneously at 1.5 MYA. Recent peaks of the TIR-*Sola* and the LINE-*R1* invasions were observed at 0.2 and 0.8 MYA, respectively.

### 3.3. TE Insertions in and near Defensome Genes Involved in Insecticide Resistance

**Figure 3:**
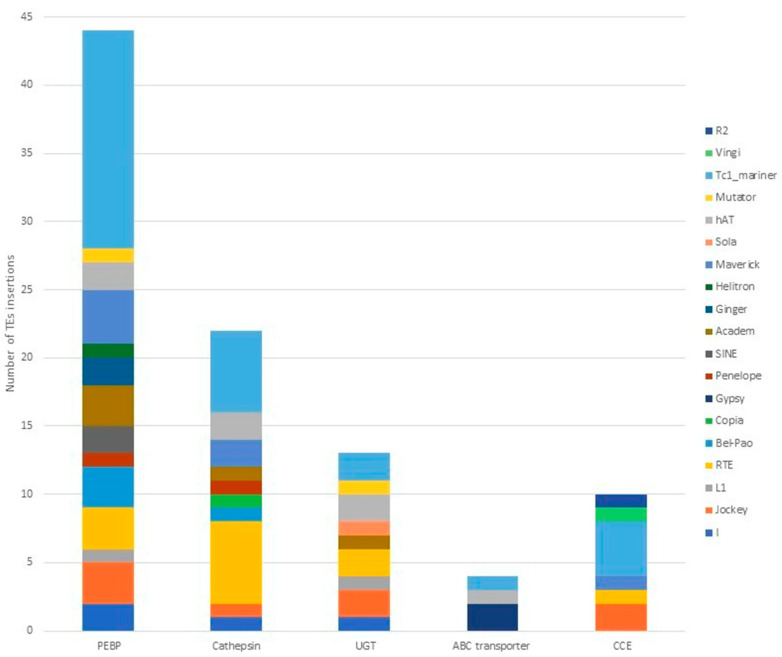
The number of the identified TE insertions in the phosphatidyl-ethanolamine binding protein (PEBP), cathepsin, UDP-Glucuronosyl-Transferase (UGT), ABC transporter, and Carboxylesterase (CCE) gene families in the *B. tabaci* genome.

The search for TE insertions in or within 2 kb near defensome genes associated with insecticide resistance revealed 94 insertion sites within or near 74 genes encoding detoxifying enzymes, of a total of 647 defensome genes identified in the *B. tabaci* genome (Table S2). The defensome genes affected by the greatest number of TE insertions are depicted in Figure 3. A total of 44 insertions were found among the 33 genes encoding phosphatidyl-ethanolamine binding protein (PEBP), including 35 insertions within the gene, five upstream and four downstream. The 21 cathepsin genes were affected by 22 insertions, including 14 insertions within the gene, one upstream and seven downstream. Moreover, the *B. tabaci* genome revealed 13 insertions among the 11 UDP-Glucuronosyl-Transferase (UGT) genes, 12 within the gene and one upstream. Four insertions were also found among ABC transporter genes, three within the gene and one downstream. Finally, 10 TEs insertions were identified within the five Carboxylesterase genes (CCE) with the exception of one insertion 270 bp upstream of the CCE gene. The involved TEs are members of *Tc1-mariner*, *hAT*, *RTE*, *Copia*, *Academ*, *Maverick*, *Mutator*, *Gypsy*, *Bel-Pao*, *Jockey*, *Sola*, *I*, *L1*, *Penelope*, and *Helitron* superfamilies.

**Figure 4:**
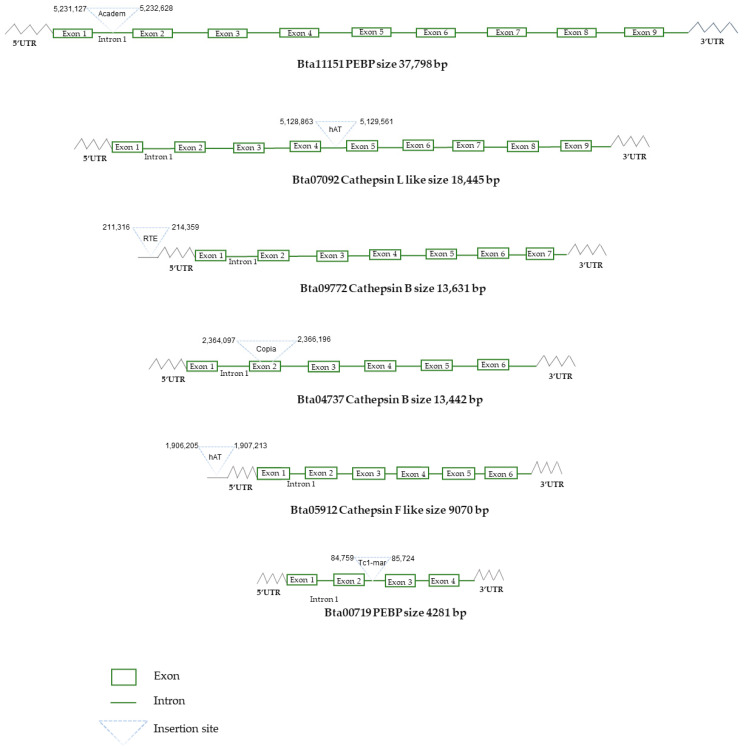
Schematic representation of the TE insertions within the six differentially expressed defensome genes in *B. tabaci*.

Since Chen et al. (2016) identified 238 defensome genes that are differentially expressed in response to insecticide treatment in a prior investigation [6], TE insertions have been searched for among these differentially expressed genes (DEGs). Results revealed six TE insertions within or upstream of the DEGs (Figure 4). These TE insertions within DEG genes involved *Tc1-mariner* and *Academ* in PEBP genes, *Copia* in the cathepsin B gene, covering all the second exon and parts of the flanking introns, and *hAT* in the cathepsin L-Like gene (Figure 4). In addition, insertions in upstream genes involved *hAT* and *RTE*, which were inserted close to the cathepsin F-Like gene and the cathepsin B gene, respectively (Figure 4).

## 4. Discussion

The current study examined the TEs’ content, in addition to their insertions, within and near the defensome genes associated with insecticide resistance in the sequenced genome of the whitefly *B. tabaci*. TE characterization is an important challenge because TEs represent a significant proportion of the genome of many species and may play a role in several regulatory, transcriptional, and protein innovations. TEs are also associated with many cases of insecticide resistance acquisition, allowing the adaptation of several insect species to environmental stressors. Fortunately, a growing number of methodologies and software tools, such as TEdenovo, LTRdigest, and HelitronScanner, have been developed to address this need [37]. In this study, we used the REPET package, which combines two main pipelines: TEdenovo, which provides a full TE library for all repeats detected, and TEannot, which is used to annotate repeats. The whitefly genome is estimated to be 615 Mb in size, with TEs representing 51% of the total sequence. Similarly, TEs represent 57.17% of the *Acanthoscurria geniculata* genome, which is 7.2 Gb in size [24]. Other insect genomes with high TE proportions and similar genome size have been reported, such as the pea aphid Hemiptera *Acyrthosiphon pisum*, which has a genome size of 542 Mb and a TE content of 37.86% [24], and the silkworm *Bombyx mori*, with a TE content equivalent to 40% of the 530 Mb genome [38]. However, the blood-sucking bug Hemiptera *Rhodnius prolixus* has a very low TE content of 5.8% of its 700 Mb genome size [39]. Furthermore, the distribution of the two main classes of TEs in the *B. tabaci* genome is similar to that of *R. prolixus*, indicating that DNA transposons occur in a higher proportion than the retrotransposons [39]. However, Class I elements are much more common in *B. mori*, *Tribolium castaneum*, and *Drosophila* species, with 89%, 87%, and 67% to 93% of the mobilome, respectively [40].

According to the TE composition analysis, TIR elements (9%) are the most abundant group of TEs in the whitefly genome, followed by the LINE (3%) and the LTR (0.6%). Abundance of TEs with TIRs has also been documented in other insects from different orders, such as the Hemiptera *R. prolixus* (4% of TIR elements) and the Diptera *Mayetiola destructor* (1% of TIR elements) [39,41]. The preponderance of TIR transposons may be linked to the activity of such elements, which may rely on active copies of the order.

The potential involvement of the detected TE insertion sites in the regulation of the defensome gene conferring insecticide resistance was examined. TE insertions within and up to 2 kb upstream of the genes have been investigated, particularly for DEGs previously described by Chen et al. 2016, given that TE insertion at this distance may affect the cis regulation of gene expression [6,35]. Our results revealed six TEs insertions, among which three were inside the cathepsin B and the PEBP genes. Interestingly, a sequence of *Copia* element covered all of the second exon of the cathepsin B gene, which is involved in intracellular proteolysis. It has been suggested that the cathepsin B gene may play a role in insecticide resistance of *B tabaci* [6]. Thus, this insertion may be the source of a novel function of the cathepsin gene as it results in an mRNA [6].

The analysis of the whitefly defensome genes revealed an insertion of a *hAT* element into the cathepsin L-Like gene intron. Similar intronic insertion was recently reported in the cotton aphid *Aphis gossypii*. This non-coding insertion implies a *Jockey* element in the cytP450 6K1-like gene that was considered as a strong candidate for imparting resistance to thiamethoxam [42]. In addition, our results revealed the insertion of a *Tc1-mariner* element and an *Academ* element in the introns of the PEBP genes. These intronic insertions may be less susceptible to selection and may be successfully spliced out during mRNA processing, leaving the function of the relevant defensome gene unaffected [43]. 

Conversely, non-coding insertions may be impacted by other regulatory mechanisms; for example, if the introns of the insecticide resistance gene contain regulatory sequences, they may result in exon skipping, alternative splicing, or variations in expression profiles. Indeed, in maize, the insertion of the *Mutator* element into the third intron of the Knotted locus resulted in ectopic expression of the transcript in leaves of mutant plants [43]. Moreover, in the *B. tabaci* genome, TEs were inserted upstream of cathepsin F-Like and cathepsin B insecticide resistance genes. Similar insertions close to the gene have been reported in *D. melanogaster*, involving the *Accord* LTR retrotransposon upstream of the Cyp6g1 [44].

The identified TE insertions may mediate insecticide resistance in *B. tabaci* through cis regulation of insecticide genes, enabling this hemipteran pest to adapt to xenobiotic stress. It is worth mentioning that TE insertions themselves contribute directly to gene evolution, as previously reported for the genes encoding SSGP proteins, which evolved by transposition of MITEs and *Ty3/gypsy* elements [45,46].

Additionally, TE age estimation revealed recent transposition events of TE elements belonging to the TIR and LINE orders, which may explain the abundance of these elements in the *B. tabaci* genome. This reactivation of TEs would have occurred concurrently with the invasion of new variants involving complete copies, which could have acted in trans to mobilize the TEs of other lineages belonging to the same group [47,48].

## 5. Conclusions

In this study, we conducted a genome-wide screening of TEs in the hemipteran pest, *B. tabaci*, which demonstrated a wide range of TE diversity, with different age distributions and activities. Some elements were inserted within and near the defensome genes involved in insecticide resistance, which may be important for *B. tabaci* to adapt. Therefore, these analyses are an important first step toward more in-depth studies that focus on promoter, end of transcription, and splicing signals deriving from TEs, in order to better estimate their impact on this pest’s insecticide resistance genes.

## Figures and Tables

**Table 1:** Summary of the identified and annotated TEs in the *B. tabaci* genome.

Classes	Genome Covrage %	Order	Superfamily	Number of Copies	Number of Lineages	Number of FLC	% of TE
Class I	5.70	LARD		859	6	14	0.066
		LINE					
			*R2*	2327	12	22	0.180
			*R1*	1015	23	50	0.079
			*RTE*	14,232	34	211	1.101
			*Jockey*	4909	87	202	0.380
			*L1*	170	1	2	0.013
			*I*	931	13	27	0.072
			*Vingi*	108	2	3	0.008
			*Hope*	24	1	3	0.002
			*Waldo*	197	3	5	0.015
			*SART*	696	14	25	0.054
			*Nimb*	952	7	11	0.074
			Loa	3	62	5	0.000
			*Unclassified*	17,698	64	179	1.369
		LTR					
			*Copia*	3726	56	99	0.288
			*Gypsy*	3797	64	147	0.294
			*Bel-Pao*	143	64	101	0.011
		PLE	*Penelope*	6993	27	52	0.541
		SINE					
			*5S*	385	2	3	0.030
			*Unclassified*	2634	40	112	0.204
		TRIM		11,424	117	319	0.884
Class II	16.55	TIR					
			*Tc1-Mariner*	5038	52	407	0.390
			*hAT*	5797	35	110	0.449
			*Mutator*	1640	46	124	0.127
			*Merlin*	23	1	3	0.002
			*Transib*	52	2	3	0.004
			*P*	135	7	10	0.010
			*PiggyBac*	117	2	4	0.009
			*PIF-Harbinger*	6	1	1	0.000
			*Ginger*	292	5	27	0.023
			*CACTA*	134	6	9	0.010
			*Academ*	1097	9	42	0.085
			*Sola*	93	2	9	0.007
			*Unclassified*	106,644	566	5239	8.252
		Crypton	*Crypton*	8	1	2	0.001
		Helitron	*Helitron*	1723	21	95	0.133
		Maverick	*Maverick*	6723	28	51	0.520
		MITE		84,436	564	9393	6.533
UnCategorized	28.81			1,005,212	2825	10,478	77.779
Total	51.06			1,292,393	4872	27,599	100

## Data Availability

The data presented in this study are available in article or Supplementary Materials.

## Supplementary Materials

The following supporting information can be downloaded at: https://www.mdpi.com/article/10.3390/insects13050396/s1, Figure S1: The age distribution in million years ago of the TEs from the *B. tabaci* genome; Table S1: Characteristics of TEs identified and annotated in the *B. tabaci* genome; Table S2: Summary of TE insertions in and near defensome genes involved in insecticide resistance in the *B. tabaci* genome; File S1: All the Full-Length Copy sequences (fasta format); File S2: Source datasets used for TE analysis (annotation of TEs in the *B. tabaci* genome, estimation of TE age distribution, TE insertions in and near defensome genes involved in insecticide resistance); Genes from detoxification and other interesting families that are differentially expressed upon insecticide treatment; TE package Insertion/Deletion Dynamics for Transposable Elements; GFF file of *B. tabaci* genes.
