# Supplementary material for: Genome-Wide Screening of Transposable Elements in the Whitefly, Bemisia tabaci (Hemiptera: Aleyrodidae), Revealed Insertions with Potential Insecticide Resistance Implications
Source: Insects. 2022 Apr 19;13(5):396. doi: 10.3390/insects13050396 (PMC9143410; doi:10.3390/insects13050396)
Supplement: Supplementary file 1 [file insects-13-00396-s001.zip › insects-1659198-proofed-s - new/Figure S1.pdf]

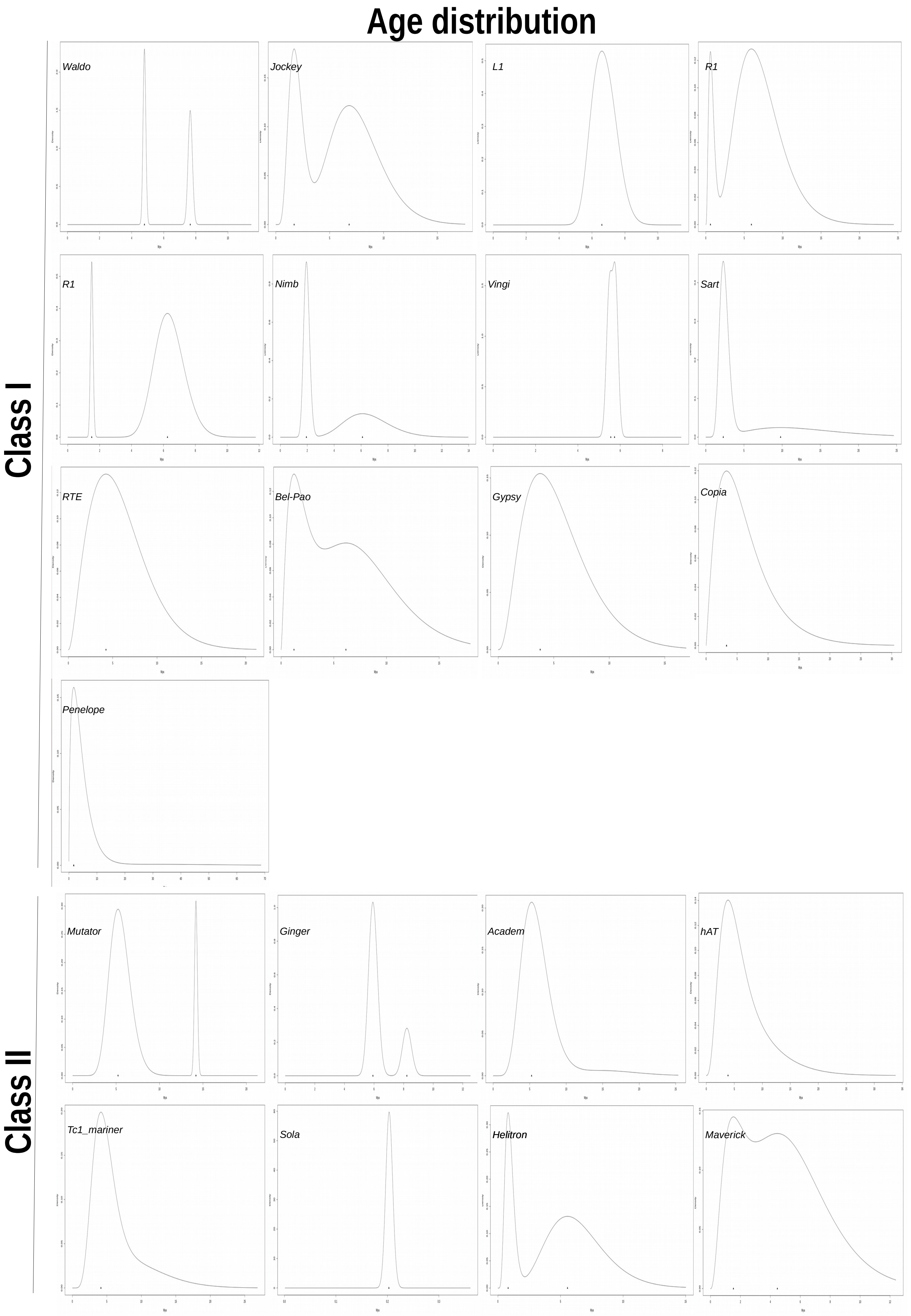

Figure S1. The age distribution in million years ago of the TEs from the B. tabaci genome. Each curve was obtained by using the TE package implemented in R and represents the estimated age distribution of the members of a single Superfamily. Gray triangles on the x-axis indicate the peak locations.
