# Supplementary material for: Genome-Wide Screening of Transposable Elements in the Whitefly, Bemisia tabaci (Hemiptera: Aleyrodidae), Revealed Insertions with Potential Insecticide Resistance Implications
Source: Insects. 2022 Apr 19;13(5):396. doi: 10.3390/insects13050396 (PMC9143410; doi:10.3390/insects13050396)
Supplement: Supplementary file 1 [file insects-13-00396-s001.zip › insects-1659198-proofed-s - new/File S2/TE package.pdf]

Package 'TE'

August 22, 2018

Type Package

Version 0.3-0

Title Insertion/Deletion Dynamics for Transposable Elements

License MIT + file LICENSE

Description Provides functions to estimate the insertion and deletion rates of transposable element (TE) families. The estimation of insertion rate consists of an improved estimate of the age distribution that takes into account random mutations, and an adjustment by the deletion rate. A hypothesis test for a uniform insertion rate is also implemented. This package implements the methods proposed in Dai et al (2018).

LazyData true

Depends R (>= 2.10)

Imports MASS, rainbow

RoxygenNote 6.1.0

NeedsCompilation no

Author Xiongtao Dai [aut, cre, cph],
Hao Wang [aut],
Jan Dvorak [ctb],
Jeffrey Bennetzen [ctb],
Hans-Georg Mueller [ctb]

Maintainer Xiongtao Dai <xdai@iastate.edu>

Repository CRAN

Date/Publication 2018-08-22 19:40:06 UTC

R topics documented:

AetLTR 2
AlyLTR 3
EstDynamics 3
MasterGene 5
MatrixModel 7
nbLackOfFitKL 8

PlotFamilies 8
print.TEfit 9
SensitivityPlot 10
TE 10

Index **12**

`AetLTR` *LTR retrotransposons in* Aegilops tauschii

Description

This data file contains the LTR retrotransposons in *Ae. tauschii*.

Format

A data frame with 18024 rows and 12 columns. Each row corresponds to a unique LTR retrotransposon, and each column corresponds to a feature of the LTR-RT. The columns are:

SeqID LTR retrotransposon sequence ID

UngapedLen Length of each LTR

Mismatch Number of mismatches

Distance Divergence, as defined by (# of mismatches) / (LTR length)

Chr Chromosome number

Start Start location in bp

Stop Ending location in bp

GroupID LTR retrotransposon Family ID

sup Super family membership

recRt5 Recombination rate

nearOld Whether the LTR-RT is near a gene that is colinear with wild emmer (TRUE) or not (FALSE)

cCodon Whether the LTR-RT is near the start codon (1) or not (-1)

logDist Log distance to the nearest gene in bp

distToGene Distance to the nearest gene in bp

References

Luo, Ming-Cheng, et al. (2017) "Genome sequence of the progenitor of the wheat D genome Aegilops tauschii." Nature 551.7681.

Dvorak, J., L. Wang, T. Zhu, C. M. Jorgensen, K. R. Deal et al., (2018) "Structural variation and rates of genome evolution in the grass family seen through comparison of sequences of genomes greatly differing in size". The Plant Journal 95: 487-503.

Dai, X., Wang, H., Dvorak, J., Bennetzen, J., Mueller, H.-G. (2018). "Birth and Death of LTR Retrotransposons in Aegilops tauschii". Genetics

`AlyLTR` *LTR retrotransposons in* Arabidopsis lyrata

Description

This data file contains the LTR retrotransposons in *Arabidopsis lyrata*.

Format

A data frame with 397 rows and 7 columns. Each row corresponds to a unique LTR retrotransposon, and each column corresponds to a feature of the LTR-RT. The columns are:

SeqID LTR retrotransposon sequence ID

UngapedLen Length of each LTR

Mismatch Number of mismatches

Distance Divergence, as defined by (# of mismatches) / (LTR length)

sup Super family membership

GroupID LTR retrotransposon Family ID

thaID Family name matched in the LTR-RT families of A. thaliana

References

Lamesch, Philippe, Tanya Z. Berardini, Donghui Li, David Swarbreck, Christopher Wilks, Rajkumar Sasidharan, Robert Muller et al. "The Arabidopsis Information Resource (TAIR): improved gene annotation and new tools." Nucleic acids research 40, no. D1 (2011): D1202-D1210.

Dai, X., Wang, H., Dvorak, J., Bennetzen, J., Mueller, H.-G. (2018+). "Birth and Death of LTR Retrotransposons in Aegilops tauschii"

`EstDynamics` *Estimate TE dynamics using mismatch data*

Description

Given the number of mismatches and element lengths for an LTR retrotransposon family, estimate the age distribution, insertion rate, and deletion rates.

Usage

```
EstDynamics(mismatch, len, r = 0.013, perturb = 2, rateRange = NULL,
  plotFit = FALSE, plotSensitivity = FALSE, pause = plotFit &&
  plotSensitivity, main = sprintf("n = %d", n))

EstDynamics2(mismatch, len, r = 0.013, nTrial = 10L, perturb = 2,
  rateRange = NULL, plotFit = FALSE, plotSensitivity = FALSE,
  pause = plotFit && plotSensitivity, ...)
```

Arguments

`mismatch`	A vector containing the number of mismatches.
`len`	A vector containing the length of each element.
`r`	Mutation rate (substitutions/(million year * site)) used in the calculation.
`perturb`	A scalar multiple to perturb the estimated death rate from the null hypothesis estimate. Used to generate the sensitivity analysis.
`rateRange`	A vector of death rates, an alternative to `perturb` for specifying the death rates.
`plotFit`	Whether to plot the distribution fits.
`plotSensitivity`	Whether to plot the sensitivity analysis.
`pause`	Whether to pause after each plot.
`main`	The title for the plot.
`nTrial`	The number of starting points for searching for the MLE.
`...`	Pass to EstDynamics

Details

`EstDynamics` estimates the TE dynamics through fitting a negative binomial fit to the mismatch data, while `EstDynamics2` uses a mixture model. For detailed implementation see References.

Value

`EstDynamics` returns a `TEfit` object, containing the following fields, where the unit for time is million years ago (Mya):

`pvalue`	The p-value for testing H_0: The insertion rate is uniform over time.
`ageDist`	A list containing the estimated age distributions.
`insRt`	A list containing the estimated insertion rates.
`agePeakLoc`	The maximum point (in age) of the age distribution.
`insPeakLoc`	The maximum point (in time) of the insertion rate.
`estimates`	The parameter estimates from fitting the distributions; see References
`sensitivity`	A list containing the results for the sensitivity analysis, with fields `time`: time points; `delRateRange`: A vector for the range of deletion rates; `insRange`: A matrix whose columns contain the insertion rates under different scenarios.
`n`	The sample size.
`meanLen`	The mean of element length.
`meanDiv`	The mean of divergence.
`KDE`	A list containing the kernel density estimate for the mismatch data.
`logLik`	The log-likelihoods of the parametric fits.

This function returns a `TEfit2` object, containing all the above fields for `TEfit` and the following:

`estimates2`	The parameter estimates from fitting the mixture distribution.

ageDist2	The estimated age distribution from fitting the mixture distribution.
insRt2	The estimated insertion rate from fitting the mixture distribution.
agePeakLoc2	Maximum point(s) for the age distribution.
insPeakLoc2	Maximum point(s) for the insertion rate.

References

Dai, X., Wang, H., Dvorak, J., Bennetzen, J., Mueller, H.-G. (2018). "Birth and Death of LTR Retrotransposons in Aegilops tauschii". Genetics

Examples

```
# Analyze Gypsy family 24 (Nusif)
data(AetLTR)
dat <- subset(AetLTR, GroupID == 24 & !is.na(Chr))
set.seed(1)
res1 <- EstDynamics(dat$Mismatch, dat$UngapedLen, plotFit=TRUE, plotSensitivity=FALSE, pause=FALSE)

# p-value for testing a uniform insertion rate
res1$pvalue


# Use a mixture distribution to improve fit
res2 <- EstDynamics2(dat$Mismatch, dat$UngapedLen, plotFit=TRUE)

# A larger number of trials is recommended to achieve the global MLE
## Not run:
res3 <- EstDynamics2(dat$Mismatch, dat$UngapedLen, plotFit=TRUE, nTrial=1000L)

## End(Not run)
```

MasterGene *Implements the master gene model in Marchani et al (2009)*

Description

Implements the master gene model in Marchani et al (2009)

Usage

```
MasterGene(mismatch, len, r = 0.013, plotFit = FALSE,
  main = sprintf("n = %d", n))
```

Arguments

`mismatch`	A vector containing the number of mismatches.
`len`	A vector containing the length of each element.
`r`	Mutation rate (substitutions/(million year * site)) used in the calculation.
`plotFit`	Whether to plot the distribution fits.
`main`	The title for the plot.

Details

For the method implemented see References.

Value

This function returns various parameter estimates described in Marchani et al (2009), containing the following fields. The unit for time is million years ago (mya):

`B`	The constant insertion rate
`q`	The constant excision rate
`lam`	The population growth rate
`R`	The ratio of the number of elements in class j over class j+1, which is constant by assumption
`age1`	The age of the system under model 1 (lambda > 1)
`age2`	The age of the system under model 2 (an initial burst followed by stasis lambda = 1)

References

Marchani, Elizabeth E., Jinchuan Xing, David J. Witherspoon, Lynn B. Jorde, and Alan R. Rogers. "Estimating the age of retrotransposon subfamilies using maximum likelihood." Genomics 94, no. 1 (2009): 78-82.

Examples

```
# Analyze Gypsy family 24 (Nusif)
data(AetLTR)
dat <- subset(AetLTR, GroupID == 24 & !is.na(Chr))
res2 <- MasterGene(dat$Mismatch, dat$UngapedLen, plotFit=TRUE)
```

MatrixModel *Implements the matrix model in Promislow et al (1999)*

Description

Implements the matrix model in Promislow et al (1999)

Usage

```
MatrixModel(mismatch, len, nsolo, r = 0.013, plotFit = FALSE,
  main = sprintf("n = %d", n))
```

Arguments

mismatch	A vector containing the number of mismatches.
len	A vector containing the length of each element.
nsolo	An integer giving the number of solo elements.
r	Mutation rate (substitutions/(million year * site)) used in the calculation.
plotFit	Whether to plot the distribution fits.
main	The title for the plot.

Details

For the method implemented see References.

Value

This function returns various parameter estimates described in Promislow et al. (1999), containing the following fields. The unit for time is million years ago (Mya):

B	The constant insertion rate
q	The constant excision rate
lam	The population growth rate
R	The ratio of the number of elements in class j over class j+1, which is constant by assumption
age1	The age of the system under model 1 (lambda > 1)
age2	The age of the system under model 2 (an initial burst followed by stasis lambda = 1)

References

Promislow, D., Jordan, K. and McDonald, J. "Genomic demography: a life-history analysis of transposable element evolution." Proceedings of the Royal Society of London B: Biological Sciences 266, no. 1428 (1999): 1555-1560.

Examples

```
# Analyze Gypsy family 24 (Nusif)
data(AetLTR)
dat <- subset(AetLTR, GroupID == 24 & !is.na(Chr))
res1 <- MatrixModel(dat$Mismatch, dat$UngapedLen, nsolo=450, plotFit=TRUE)
```

`nbLackOfFitKL` *Calcualte the KL divergence of a negative binomial fit to the mismatch data.*

Description

Calcualte the KL divergence of a negative binomial fit to the mismatch data.

Usage

```
nbLackOfFitKL(res)
```

Arguments

`res`	A TEfit object.

Examples

```
# Analyze Gypsy family 24 (Nusif)
data(AetLTR)
dat <- subset(AetLTR, GroupID == 24 & !is.na(Chr))
set.seed(1)
res1 <- EstDynamics(dat$Mismatch, dat$UngapedLen, plotFit=TRUE, plotSensitivity=FALSE, pause=FALSE)
nbLackOfFitKL(res1)
```

`PlotFamilies` *Plot the age distributions or insertion rates for multiple families.*

Description

Plot the age distributions or insertion rates for multiple families.

Usage

```
PlotFamilies(resList, type = c("insRt", "ageDist"), ...)
```

Arguments

`resList`	A list of TEfit/TEfit2 objects, which can be mixed
`type`	Whether to plot the insertion rates ('insRt') or the age distributions ('ageDist').
`...`	Passed into plotting functions.

Value

A list of line data (plotDat) and peak locations (peakDat).

Examples

```
data(AetLTR)
copia3 <- subset(AetLTR, GroupID == 3 & !is.na(Chr))
gypsy24 <- subset(AetLTR, GroupID == 24 & !is.na(Chr))
res3 <- EstDynamics(copia3$Mismatch, copia3$UngapedLen)
res24 <- EstDynamics2(gypsy24$Mismatch, gypsy24$UngapedLen)

# Plot insertion rates
PlotFamilies(list(`Copia 3`=res3, `Gypsy 24`=res24))

# Plot age distributions
PlotFamilies(list(`Copia 3`=res3, `Gypsy 24`=res24), type='ageDist')
```

---

print.TEfit *Print a TEfit or TEfit2 object*

Description

Print a TEfit or TEfit2 object

Usage

```
## S3 method for class 'TEfit'
print(x, ...)

## S3 method for class 'TEfit2'
print(x, ...)
```

Arguments

x	A TEfit or TEfit2 object
...	Not used

SensitivityPlot *Generate sensitivity plots*

Description

Create sensitivity plots of a few families to investigate different death rate scenarios

Usage

```
SensitivityPlot(resList, col, xMax, markHalfPeak = FALSE,
  famLegend = TRUE, rLegend = names(resList), ...)
```

Arguments

resList	A list of families returned by EstDynamics
col	A vector of colors
xMax	The maximum of the x-axis
markHalfPeak	Whether to mark the time points with half-intensity
famLegend	Whether to create legend for families
rLegend	Text for the legend for families
...	Passed into matplot

Examples

```
data(AetLTR)
copia3 <- subset(AetLTR, GroupID == 3 & !is.na(Chr))
copia9 <- subset(AetLTR, GroupID == 9 & !is.na(Chr))
res3 <- EstDynamics(copia3$Mismatch, copia3$UngapedLen)
res9 <- EstDynamics(copia9$Mismatch, copia9$UngapedLen)
SensitivityPlot(list(`Copia 3`=res3, `Copia 9`=res9))
```

TE *TE: Insertion/Deletion Dynamics for Transposable Elements*

Description

TE package for analyzing insertion/deletion dynamics for transposable elements

Details

Provides functions to estimate the insertion and deletion rates of transposable element (TE) families. The estimation of insertion rate consists of an improved estimate of the age distribution that takes into account random mutations, and an adjustment by the deletion rate. This package includes functions `EstDynamics` and `EstDynamics2` for analyzing the TE divergence, and visualization functions such as `PlotFamilies` and `SensitivityPlot`. This package implements the methods proposed in Dai et al (2018+).

Author(s)

Xiongtao Dai <xdai@iastate.edu>, Hao Wang Jan Dvorak Jeffrey Bennetzen Hans-Georg Mueller

Maintainer: Xiongtao Dai <xdai@iastate.edu>

References

Luo, Ming-Cheng, et al. (2017) "Genome sequence of the progenitor of the wheat D genome Aegilops tauschii." Nature 551.7681.

Dai, X., Wang, H., Dvorak, J., Bennetzen, J., Mueller, H.-G. (2018). "Birth and Death of LTR Retrotransposons in Aegilops tauschii". Genetics

Index

AetLTR, 2
AlyLTR, 3

EstDynamics, 3, *10*
EstDynamics2 *(*EstDynamics*)*, 3

MasterGene, 5
MatrixModel, 7

nbLackOfFitKL, 8

PlotFamilies, 8
print.TEfit, 9
print.TEfit2 *(*print.TEfit*)*, 9

SensitivityPlot, 10

TE, 10
TE-package *(*TE*)*, 10
